# Potential Therapeutic Effects of Meditation for Treating Affective Dysregulation

**DOI:** 10.1155/2014/402718

**Published:** 2014-08-12

**Authors:** Natalie T. Y. Leung, Mandy M. Lo, Tatia M. C. Lee

**Affiliations:** ^1^Laboratory of Neuropsychology, The University of Hong Kong, Hong Kong; ^2^Laboratory of Cognitive Affective Neuroscience, The University of Hong Kong, Hong Kong; ^3^Institute of Clinical Neuropsychology, The University of Hong Kong, Hong Kong

## Abstract

Affective dysregulation is at the root of many psychopathologies, including stress induced disorders, anxiety disorders, and depression. The root of these disorders appears to be an attenuated, top-down cognitive control from the prefrontal cortices over the maladaptive subcortical emotional processing. A form of mental training, long-term meditation practice can trigger meditation-specific neuroplastic changes in the brain regions underlying cognitive control and affective regulation, suggesting that meditation can act as a kind of mental exercise to foster affective regulation and possibly a cost-effective intervention in mood disorders. Increasing research has suggested that the cultivation of awareness and acceptance along with a nonjudgmental attitude via meditation promotes adaptive affective regulation. This review examined the concepts of affective regulation and meditation and discussed behavioral and neural evidence of the potential clinical application of meditation. Lately, there has been a growing trend toward incorporating the “mindfulness” component into existing psychotherapeutic treatment. Promising results have been observed thus far. Future studies may consider exploring the possibility of integrating the element of “compassion” into current psychotherapeutic approaches.

## 1. Introduction

Affective regulation is fundamental to adaptive social and psychological functioning, and affective dysregulation is the root of a number of mental disorders, including maladaptive stress reactions, anxiety, and depression. Associated with these disorders is significant maladaptive functioning, which creates heavy socioeconomic costs for societies worldwide. For example, major depressive disorder (MDD) ranks second in the global burden of disease [1]. Lifetime prevalence rate of MDD is relatively higher for women than men, suggesting a sex-related difference in affective regulation [2]. Hence, there is a timely demand for cost-effective intervention in disorders stemming from affective dysregulation. Recently, in addition to the pharmaceutical advancement in Eastern and Western medicine in treating affective disorders, people are yearning for cost-effective behavioral strategies that help prevent and intervene in affective dysregulation and can be incorporated into large-scale public health programs. One promising candidate that has been well received by people across ethnic and cultural groups is meditation. A literature review reveals reports of the therapeutic effects of meditation on depression [3, 4] and grieving [5].

This review covers the existing empirical evidence of the neural effects of mindfulness and compassion meditation. While mindfulness meditation has received most of the empirical attention [6, 7], there has been growing interest in compassion meditation, which emphasizes positive affective states. To the best of our knowledge, reviews of the empirical evidence of compassion meditation, especially its neural effects, are scarce. We intend to bridge this gap by discussing the effects of compassion meditation and mindfulness meditation on the brain and behaviors, as well as their potential therapeutic value in treating some commonly identified disorders stemming from affective dysregulation (e.g., stress induced disorders, anxiety, and depression). This review will first lay the theoretical groundwork for affective regulation and meditation. It will then present evidence from behavioral and neuroimaging studies to corroborate the important role of meditation—specifically, mindfulness meditation and compassion meditation—in affective regulation in both healthy and clinical populations.

## 2. Affective Regulation

A spectrum of affective-regulation strategies for downregulating negative emotional responses has been identified. One that has received much research attention is the reappraisal of affective stimuli in the brain. This involves reappraising the affective value via reinterpreting the meanings of emotion-evocative stimulus in order to mitigate its affective impact [8–11]. For example, when viewing disgust-eliciting film clips, cognitive reappraisal downregulated the negative emotional experience to a greater extent when compared to expressive suppression, a response-focused strategy that targets inhibiting behaviors (e.g., facial expression) associated with emotional response [12]. Neuroimaging findings have reported that cognitive reappraisal is associated with reduced neural responses in the amygdala and insula but enhanced neural activation in the prefrontal cortices that involve cognitive control of emotion. Expressive suppression demonstrated an opposite pattern [12]. Stemming from the concept of experience-induced neuroplastic change [13, 14], an experience specifically designed and induced to the brain to facilitate adaptive cognitive reappraisal may promote affective regulation by downregulating the negative emotional experience of affective stimuli. The human brain is a malleable organ whose structure and functions are changeable in response to experience and learning [15, 16].

## 3. Meditation and Affective Regulation

Empirical findings demonstrated that a regular practice of meditation induces significant changes in brain structure and functions; therefore, behaviors change. Meditation is a mental exercise of regulatory practices intending to attain certain psychological states that involve mind and body interaction of being in the present moment with a nonjudgmental attitude [17]. The aim is to cultivate specific psychological states or mental capacities, such as quiescence and equanimity [6, 18]. Quiescence is to be tranquil at rest—a completely relaxed bodily state with full concentration on the present moment [19]. Equanimity, on the other hand, is the ability to accept things as they are, with a balanced state of mind that is calm and spacious. This enables one to be present, fully and nonjudgmentally [18, 20, 21].

There is a vast array of contemplative practices and techniques within the Buddhist tradition. Our review intends to focus exclusively on two specific forms of meditation which, from the perspective of neuroscience, engage in different cognitive processes. Mindfulness meditation is a form of meditation that emphasizes on monitoring the present experience from moment to moment while acknowledging the nature or content of the experience without spontaneously reacting to it [6]. For this reason, the practice of mindfulness meditation highly hinges on the ability to control attention, which is essential for the practitioners to openly monitor all the experience in the present moment without focusing their attention on an explicit object. On the contrary, the ability to regulate emotions is more fundamental to compassion meditation or loving-kindness meditation (LKM) in general. When viewing others' suffering, it is not uncommon for us to feel distress or upset about others' painful experience. The practice of LKM, which involves cultivation of a state of universal love and compassion towards self and other beings with a desire to relieve their pain and suffering [20, 22], can attenuate the emotional impact by sharpening one's ability to downregulate the negative emotions (e.g., sadness) triggered by distressful experience and restore the emotional balance.

Researchers have made significant progress in understanding how meditation promotes positive emotions. Using a high-resolution EEG to measure neural oscillations, researchers have reported patterns of brain electrical activity associated with a positive, “blissful” experience [23]. Moreover, functional neuroimaging findings have suggested that meditation could increase neural activity associated with positive emotions [24]. Researchers proposed that the affect-labeling technique that meditators use is the neurocognitive mechanism that reduces negative affect [25].

While meditation emphasizes nonjudgmental attention to a present moment experience, cognitive reappraisal focuses primarily on reinterpreting the meaning of an emotion-eliciting stimulus. A growing body of research has shown that these two theoretical constructs are interrelated to exercise their effects on affective regulation at both neural and behavioral levels.

### 3.1. Behavioral Evidence

Several behavioral studies reported significantly better affective regulation for meditators than nonmeditators [2, 26]. For instance, Lykins and Baer [26] compared the differences between experienced meditators in mindfulness meditation and demographically similar nonmeditators and found that the two groups significantly differ on several aspects, including higher scores of several adaptive psychological functions and lower scores of several maladaptive emotion traits and cognitive-related variables on experienced meditators over nonmeditators. This observation suggests that individuals who practice mindfulness meditation regularly are more ready to observe thoughts and feelings nonjudgmentally, ruminate less on emotion, and are more likely to function adaptively in emotionally arousing situations. They concluded that the practice of mindfulness meditation cultivates mindfulness in daily life, therefore promoting psychological well-being.

Kemeny et al. [27] studied the relationship between mindfulness meditation and psychological functioning in a sample of 82 healthy females randomly assigned to the training group or wait-list control group. This 42-hour training program (4 full days and 4 evening sessions over 8 weeks) combined mindfulness meditation practices with affective regulation strategies. They found significantly decreased negative affect (specifically, ruminative thoughts), reduced destructive emotions and emotional behavior, and higher acceptance and awareness of emotions and feelings in the training group as compared to the control group. The psychological benefits were maintained for 5 months after completion of the training program, which demonstrated the long-term effects of intensive meditation training in healthy individuals. Arch and Craske [28] found that participants, even after a brief mindfulness induction, displayed more adaptive affective regulation when faced with negative stimuli. The experimental group showed a greater willingness to maintain visual contact with the pictures depicting strong negative affect. This group was also relatively more stable and less emotionally volatile across affective slide types when compared to the control groups.

A number of behavioral studies attempted to investigate the effects of LKM training [29, 30]. In a recent study, 105 undergraduate women were randomized into self-compassion, attention control, or no intervention groups. After brief self-compassionate training, the self-compassion group showed greater resilience during a social threat than the other two groups [29]. A longitudinal study conducted by Fredrickson et al. [30] found that a 7-week LKM training for novice meditators significantly increased positive emotion (e.g., love, pride, hope, gratitude, and contentment). In turn, these changes enhanced life satisfaction when compared to the waitlist group. After completing a brief guided LKM and focus-attention meditation (FAM) training once a week for 12 weeks, participants in both groups showed decreased anxiety and negative affect and increased hope [31].

### 3.2. Neural Evidence

Evidence from neuroimaging studies provided further support for the beneficial role of long-term meditation practice in emotional processing. For example, long-term meditators did not show any significant increase in late positive potentials (LPP) (LPP is known to be evoked by emotionally arousing stimuli, and its amplitude increases with the emotional potency of the stimuli [32]) when viewing the negative images, as opposed to their matched controls, which had no prior experience in meditation. It appears that meditation practice can possibly enhance emotional processing and hence mitigate the affective impact of emotion-eliciting stimuli. On the other hand, Taylor et al. [33] revealed that mediators perceived pictures portraying positive and negative emotions to be less intense during a mindful (rather than nonmindful) state. Their functional neuroimaging findings further indicated that meditators recruit different neural networks when processing emotional stimuli. Farb et al. [34] revealed that mindfulness-based stress reduction (MBSR) [35] training could modulate neural reactivity in response to sadness provocation. MBSR is an 8-week intensive and well-structured program, in which mindfulness meditation is a core element, that intends to promote mindfulness or present moment awareness as well as well-being in all individuals, especially those with stress, pain, and other medical conditions [17, 35]. Similarly, Hölzel et al. [36] also documented the longitudinal neural effects of MBSR in terms of significant increases in gray matter concentration in the left hippocampus, posterior cingulate cortex, temporoparietal junction, and the cerebellum after 8 weeks of MBSR training. It indicated that mindfulness meditation could benefit memory and learning and affective regulation, as well as social cognition.

Relatively few studies have shed light on the neural effect of LKM or compassion meditation when compared to mindfulness meditation. During voluntary cultivation of compassion states, expert LKM meditators manifested stronger neural activity in the anterior insula when responding to negative sounds relative to positive sounds, suggesting that LKM practice can sharpen an individual's ability to identify others' emotional states based on their voice [20]. Compassion meditation training can also alleviate the emotional impact of witnessing others' distress. Instead of eliciting activations in the neural network associated with empathy for pain, Klimecki et al. [37] revealed that participants, after receiving compassion meditation training, reported an increase in positive affect and engagement in the neural regions, implicating a positive affect and affiliation in response to others' suffering. Findings from longitudinal studies also found that short-term compassion meditation training can promote altruistic behavior and increase the tendency to approach, rather than avoid, the suffering of others [38]. After receiving two weeks of compassion or reappraisal training, participants' altruistic behavior, as measured by greater redistribution of funds toward victims of unfair treatment, was positively associated with neural activation in regions implicating social cognition and affective regulation, including the inferior parietal cortex and dorsolateral prefrontal cortex. This significant correlation was specific to the participants who underwent compassion training but not their counterparts from the reappraisal training group, suggesting a training-specific change [38]. Another study also reported that 8 weeks of cognitive-based compassion training could significantly enhance the accuracy in inferring others' mental states in the Reading the Mind in the Eyes Test [39] and increased the neural activation in the inferior frontal gyrus and dorsomedial prefrontal cortex, which are important for the theory of mind [40]. Taken together, these findings suggest that functional neuroplasticity is not unique to cognitive or motor domains, which raises the possibility of inducing neural plastic changes in the affective regulation system, assuaging the impact of affective dysregulation. Further research will be needed to explore this plausibility.

## 4. Potential Clinical Applications of Meditation

Findings from recent studies suggest that different forms of meditation induce meditation-specific effects on the brain. Lee et al. [22] compared the neural effects of FAM and LKM. Their findings revealed that only FAM is associated with different patterns of activation in the attention-related region. Furthermore, FAM and LKM practitioners engage in different neural networks when processing affective stimuli. The practices of FAM and LKM are associated with different patterns of neural activity in regions involved in attention-related processing and in emotion processing and regulation, respectively. Meditation-induced neuroplastic changes are not limited to functional activity. Meditation may modify brain morphometry. For example, Leung et al. [41] observed that long-term LKM practitioners are associated with enlarged right angular and posterior parahippocampal gyri, regions heavily involved in empathy and affective regulation. These promising findings shed light on the plausibility of designing a specific mental exercise (e.g., meditation) to enhance a particular cognitive or affective functioning, which can have consequential implications for the clinical population, especially those suffering from affective disorders. There is a growing trend toward integrating the core elements of mindfulness meditation with the current psychotherapeutic approach. In subsequent sections, we will review the empirical evidence of the effectiveness of meditation as a form of treatment in the clinical population.

### 4.1. Stress Induced Disorder

Prolonged exposure to stress increases the risk of developing physical and mental illnesses. Substantial evidence has shown that stress-induced responses can be ameliorated by mindfulness meditation. Following the MBSR program, the level of self-perceived stress dropped significantly among the individuals who initially reported a high level of stress right before participating in the MBSR program. Changes in self-perceived stress level induced by MBSR were also found to be positively correlated with the morphological changes in the right amygdala. In other words, the more the stress level decreased, the greater the gray matter density of the right amygdala decreased, suggesting that mindfulness meditation can induce changes in both behavioral and neural levels [42].

### 4.2. Anxiety Disorders

In another line of research, focus has shifted to investigating the potential value of MBSR in alleviating anxiety symptoms among patients with social phobia/social anxiety disorder (SAD). A few studies have examined the neural effect of MBSR in patients with SAD. Patients with SAD, after completing MBSR, demonstrated a reduction in neural activity in the amygdala and an increase in neural activity in attention-related regions when performing a breath-focused attention task [43]. In a later study, Goldin et al. [44] included a control group (aerobic exercise stress reduction program (AE)) and compared its effectiveness with that of MBSR among patients with SAD. Their findings showed that self-reported emotional reactivity to negative self-beliefs decreases to a similar extent in both MBSR and AE groups. They also identified a dissociable neural pattern between MBSR and AE groups during an affective regulation task. Specifically, neural activations in the attention-related regions increased in the MBSR but decreased in the AE group, reflecting greater engagement in neural regions involved in attention upon completion of the MBSR program.

Building on the core concept of cognitive behavioral therapy and mindfulness meditation, mindfulness-based cognitive therapy (MBCT) has been developed to treat depression (discussed below) and clinical patients with generalized anxiety disorder (GAD). Preliminary findings revealed a reduction in self-reported depressive and anxiety symptoms in GAD patients following MBCT [45]. However, this pilot study lacks a control group for comparison, so their results should be interpreted with caution. Further empirical investigation will be needed to verify the effectiveness of MBCT in treating GAD patients.

### 4.3. Major Depressive Disorder

Because of the biased cognitive processes in people suffering from MDD, MBCT tackles the cognitive processes that create the vulnerability to relapse in MDD patients. As one prominent feature of MDD is an inability to disengage from negative stimuli due to heightened emotional reactivity and attenuated cognitive control [46], the “mindfulness” component in the MBCT can afford the MDD patients an opportunity to cultivate awareness of their own thoughts or emotions and to approach them from a wider and decentered perspective (e.g., “I am not my thoughts”) [47]. The MBCT advocates the detached observation of thoughts or emotions without responding to the emotional stimuli spontaneously. The MBCT capitalizes on the nonjudgmental awareness cultivated by mindfulness meditation, which allows the MDD patients to observe and disengage from their negative thoughts or emotions as they arise, rather than trying to change or react to them spontaneously. The MBCT is conducted on a group basis and is currently only applicable to MDD patients who are in remission or recovery, whereas the MBSR targets individuals suffering from stress, pain, and other medical conditions. A number of studies have provided initial support for the effectiveness of MBCT in reducing the relapse rate of depression, especially for MDD patients with more than 3 previous episodes of depression [47]. Britton et al. [48] found that individuals with partially remitted depression in the MBCT group reported an overall attenuation in emotional reactivity (in terms of anxiety) after performing the Trier Social Stress Test. In contrast, similar differences were not observed in their counterparts in the waitlist control group after undergoing the same stress test. These findings suggested that MBCT could accelerate the process of recovering from negative emotion-provocative stressors and mitigate the emotional impact induced by stressful events. Thus far, the effectiveness of MBCT has been found to be comparable to that of antidepressant medication [49, 50]. Self-reported symptoms of depression were also found to be significantly reduced after receiving MBCT, whereas a similar reduction was not identified in patients who only received their usual pharmacological treatment [51]. It remains unclear whether MBCT can also benefit other clinical populations, but recent efforts have examined the potential therapeutic value of MBCT in treating patients in the acute phase of MDD and patients with bipolar disorder [52]. Its effectiveness is yet to be validated.

## 5. Discussion

Considerable evidence has documented experience-dependent neuroplastic changes in the brain structure [15, 22, 53], which raises the possibility of designing a specific kind of training directed at a particular function. Meditation, a mental exercise inducing a specific experience in the brain, is the focus of this review. A substantial body of research has evidenced that mindfulness and compassion meditation can have a positive impact on attention and affective regulation [54, 55] and can induce long-lasting morphological changes in the corresponding neural regions—specifically, the prefrontal cortices and amygdala [56–58]. These findings indicate that mindfulness or compassion meditation, as a kind of mental training in cognitive control and affective regulation, can be a potential form of intervention for mood disorders. This review explored the potential clinical application of meditation for disorders stemming from affective dysregulation by assessing behavioral and neural evidence of the relationship between meditation and emotion functioning. The core effect of meditation on promoting adaptive affective regulation is its effect on halting maladaptive automatic thoughts while allowing the opportunity to rectify cognitive and emotional biases associated with specific emotional memory and/or affective stimuli. Furthermore, through voluntary cultivation of positive emotional states [59], people suffering from MDD may learn to foster self-compassion. The outcome could be prevention of future relapse in terms of offsetting the detrimental effects brought on by negative self-referential thoughts.

To date, most research in the field of meditation has focused specifically on investigating the trait effects of meditation practice (experienced meditators versus meditation novices) and the longitudinal effects of meditation training in a normal, healthy population. Comparatively less empirical attention has been paid to the clinical application of meditation. Even though the existing studies provided initial support for the effectiveness of MBCT in relieving depressive and anxiety symptoms and preventing recurrence of depressive episodes, their interpretation is inevitably obscured by the lack of a comparison group, concurrent antidepressant medication, and a relatively short follow-up period. Future research shall direct its focus toward verifying whether MBCT can act as an alternative treatment in mood disorders, elucidating the mechanism of action initiated by meditation.

This review only probes the behavioral and neural evidence that addresses the effect of mindfulness and compassion meditation on affective regulation among healthy adults as well as people suffering from stress-induced disorders, MDD, and SAD/GAD. Although a number of studies have examined the potential value of applying meditation to patients with other psychiatric disorders (e.g., schizophrenia), this area of research is not included here as dysregulation of the affective system does not seem to contribute to the positive and negative symptoms observed in schizophrenia patients. This review will be more condensed if the discussion is restricted to affective disorders resulting directly from dysfunction in the affective regulation system.

Future studies will shed light on the possible role of meditation in assuaging grief. Thus far, only one study has examined the relationship between mindfulness meditation training and the grieving process in patients diagnosed with chronic pain [5]. It might be worthwhile for future research to explore the healing power of meditation in bereavement.

## 6. Conclusions

Adaptive affective regulation hinges on the top-down cognitive control of the prefrontal regions over the bottom-up emotional processing that is carried out in the subcortical regions. If there is an aberrant activation in the limbic system (specifically, the amygdala) and cognitive control is weakened, there will be heightened responses to emotional stimuli, which might in turn lead to the onset of mood disorders (e.g., MDD) [46]. The mainstream treatment approaches for MDD are antidepressant medication and cognitive behavioral therapy. The latter tackles the maladaptive thinking pattern (biased attention toward sad stimuli) that pervades MDD patients. In recent decades, substantial evidence from behavioral and neuroimaging studies has documented the beneficial role of meditation in promoting adaptive affective regulation. Meditation advocates a cultivation of awareness and acceptance of our thoughts and emotions with a nonjudgmental attitude, which encourages an individual to observe, rather than avoid, positive or negative thoughts and emotions from a detached view. Because of its emphasis on present-moment awareness and acceptance, a growing body of research has explored the potential clinical applications for disorders stemming from affective dysregulation. Current evidence reveals that meditation, a form of mental training and exercise, may prevent and intervene in mood and other affective disorders.
